# A middle-aged woman with cough, expectoration and fever

**DOI:** 10.4103/0256-4947.67080

**Published:** 2010

**Authors:** Vishal Sharma, Alka Sharma, Vivek Kumar

**Affiliations:** a From the Department of Medicine, University College of Medical Sciences, Delhi, India; b From the Department of Nephrology, Post Graduate Institute of Medical Education and Research, Chandigarh, India

A 35-year-old woman presented with a history of productive cough and fever for a week. The fever was high grade and associated with chills. The sputum was yellowish and viscid. The patient had a history of recurrent episodes of cough and expectoration since childhood, which had needed frequent hospitalizations. Fifteen years after her marriage, she had no children. She was a nonsmoker. On examination her blood pressure was 126/80, pulse rate was 116/minute and respiratory rate was 36/minute. She had clubbing in all her digits. Her heart sounds were best heard on the right side of the chest, and the apex was best felt in right fifth intercostal space. The examination of the chest revealed bilateral crackles and rhonchi. Her chest roentgenogram revealed dextrocardia (Figure 1). ECG showed a tall R wave in V1, an inverted P in lead I and aVL. A high-resolution CT of the chest confirmed the presence of dextrocardia and extensive bronchiectasis of the right lung and left lower lobe (Figure 2). Ultrasound of the abdomen confirmed reversal of positions of abdominal viscera. The woman was managed with intravenous antibiotics, bronchodilalors and mucolytics. She improved with therapy, with remission of fever and reduction in sputum production. She was discharged 15 days later after improvement.

**Figure 1:**
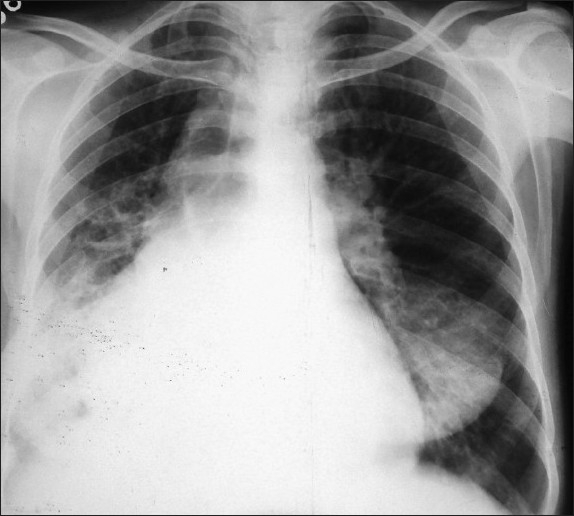
Chest roentgenogram showing dextrocardia.

**Figure 2:**
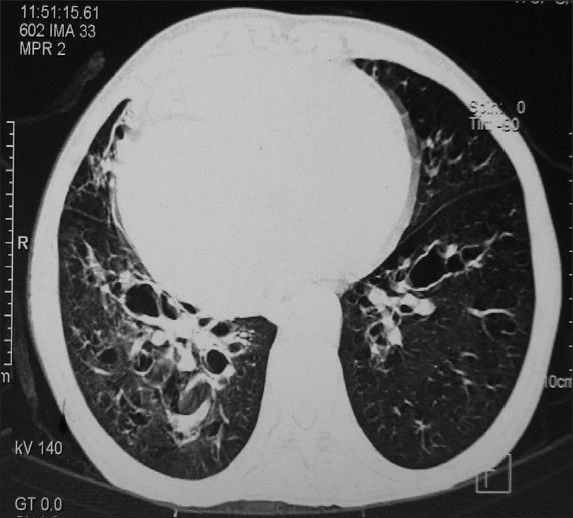
CT chest showing bronchiectasis.

What is the diagnosis?

## FOR THE ANSWER, VISIT:

http://www.saudiannals.net

